# A qualitative study of healthcare professionals’ experiences of providing maternity care for Muslim women in the UK

**DOI:** 10.1186/s12884-020-03096-3

**Published:** 2020-07-10

**Authors:** Shaima Mohamed Hassan, Conan Leavey, Jane S. Rooney, Shuby Puthussery

**Affiliations:** 1 NIHR ARC NWC, Department of Primary Care and Mental Health, University of Liverpool, Liverpool, UK; 2 Faculty of Education, Health and Community, Public Health Institute. Liverpool John Moores University, Liverpool, UK; 3 Faculty of Education, Health and Community. School of Nursing and Allied Health, Liverpool John Moores University, Liverpool, UK; 4 Maternal and Child Health Research Centre, Institute for Health Research, University of Bedfordshire, Luton, UK

**Keywords:** Maternity services, Cultural competency, Muslim women, Healthcare professionals, Exploratory study

## Abstract

**Background:**

A growing Muslim population in the UK suggests the need for healthcare professionals (HCPs) to gain a better understanding of how the Islamic faith influences health related perceptions and healthcare seeking behaviour. Although some researchers have explored the experiences of Muslim women as recipients of healthcare, little attention has been paid to the challenges HCPs face as service providers on a day-to-day basis whilst caring for Muslim women. The aim of this study was to investigate HPCs lived experiences of providing maternity care for Muslim women.

**Method:**

Data was collected through twelve semi-structured one-to-one qualitative interviews with HCPs in a large National Health Service (NHS) maternity unit located in the North West of England. Interview participants included Community and specialist clinic (e.g. clinic for non-English speakers), Midwives in a variety of specialist roles (7), Gynaecology Nurses (2), Breastfeeding Support Workers (2) and a Sonographer (1). The audio-recorded interviews were transcribed and analysed thematically.

**Results:**

The majority of participants expressed an understanding of some religious values and practices related to Muslim women, such as fasting the month of Ramadhan and that pregnant and breastfeeding women are exempt from this. However, HCPs articulated the challenges they faced when dealing with certain religious values and practices, and how they tried to respond to Muslim women’s specific needs. Emerging themes included: 1) HCPs perceptions about Muslim women; 2) HCPs understanding and awareness of religious practices; 3) HCPs approaches in addressing and supporting Muslim women’s religious needs; 4) Importance of training in providing culturally and religiously appropriate woman-centred care.

**Conclusion:**

Through this study we gained insight into the day-to-day experiences of HCPs providing care provision for Muslim women. HCPs showed an understanding of the importance of religious and cultural practices in addressing the needs of Muslim women as part of their role as maternity care providers. However, they also identified a need to develop training programmes that focus on cultural and religious practices and their impact on women’s health care needs. This will help support HCPs in overcoming the challenges faced when dealing with needs of women from different backgrounds.

## Background

There have been many improvements, over several decades, to childbirth services in the UK. These changes, emphasise the importance of a personalised and woman-centred approach to care in enhancing the experiences of women using maternity services [1–3]. The Better Birth report (2016) and the NHS long-term plan (2019) both highlight that every women and pregnancy is different [1, 3]. Consequently, the vision for maternity services is that all care is personalised around each woman’s needs, choices and decisions [1, 3]. UK maternity care policies explicitly advocate for a woman-centred approach to maternity care that is accessible, efficient and responsive to the changing needs of women, and ensures choice, access and continuity of care. Nevertheless, existing evidence suggests that women from different Black, Asian and Minority Ethnic (BAME) backgrounds in the UK continue to be at higher risk of poorer maternal health outcomes compared with women from a white background [4, 5]. For example, maternal mortality rates are five times higher for women from Black ethnic backgrounds and three times higher for women from Asian ethnic backgrounds compared with White women [5].

As such, substantial ethnic health inequalities in maternity outcomes persist in the UK coupled with maternity services that continue to struggle to provide appropriate care that meets the needs of women from diverse populations [4–8]. This is especially concerning against the background of a growing ethnically and culturally diverse population within the UK. Women from different BAME backgrounds may face multiple barriers in accessing and using maternal services including language and cultural or religious barriers. Consequently, a ‘one size fits all’ model, despite its high standards, may not meet the needs of women from BAME backgrounds [9].

Inequalities in maternal health experiences and outcomes between BAME and native groups in the UK pose a challenge to Healthcare Professionals (HCPs) and policymakers [5, 7]. It is important that HCPs and maternal services acknowledge that the choices that are made by women from all ethnic groups (such as whether and when to have children, or how many children to bear), do not occur in isolation but are socially and culturally bound [10]. Making assumptions about people from ‘other cultures’ on the basis of what is considered ‘normal’ or ‘appropriate’ to ‘us’ can lead to misunderstandings and failure to consider individual difference within groups, which limits the ability of healthcare services in addressing the need of diverse communities [4, 7, 9, 10].

Improving maternity care services for women of all backgrounds is a key priority both nationally and world-wide [1, 2, 11]. For example, the World Health Organization (WHO) supports the notion of ‘culturally-appropriate’ maternity care services that take into account a woman’s personal preferences and their cultural and value system [11]. In order to do this, it is essential that HCPs understand when and how cultural or religious context makes a difference in a woman’s experience and utilisation of maternity services and when it does not [9]. This study explores HPCs lived experiences of providing maternity care for Muslim women, who are from the second largest religious minority group in the UK [12].

Muslim communities in the UK are historically, culturally, ethnically and linguistically diverse including both immigrant Muslims and native-born Muslims [13]. This underpins the fact that Muslims in the UK do not constitute a single homogeneous group but instead a community of communities. The habits, customs, superstitions, tribal or ethnic codes of conduct of Muslims are shaped by religious values and practices [13]. Their shared religious beliefs bring about common healthcare needs and challenges including for example, the need for medications that meet Islamic lawful requirements (free from animal-based substance). A recent study, highlighted that for Muslim women in the UK religion values and practices were embedded within most aspects of the maternity experience [14]. Muslim women used their religious beliefs as a recourse to help them stay positive, optimistic and resilient when faced with struggles throughout their maternity journey. However, it can also be a source of contention and anxiety in a maternity setting especially when women feel misunderstood and stigmatised for their beliefs [14].

The diversity within the Muslim communities in the UK and their religious practices may present different challenges for some HCPs from different cultural backgrounds when providing care for Muslim women [15]. This study aimed to gain an understanding of HCPs perception of their interactions with Muslim women and the challenges they faced in providing culturally and religiously appropriate woman-centred care.

## Method

This paper presents findings from the third phase of a three-phase qualitative study that explored Muslim women’s experiences of motherhood while engaging with NHS maternity services [14, 16]. This third phase explored HCPs experiences of providing maternity services for Muslim women in the UK. Qualitative approaches are ideally suited to our research aim to further enhance our understanding of individual experiences of healthcare, health behaviour and healthcare practices and to ultimately improve the management and provision of health services for Muslim women [17]. This research approach helped us to gain valuable insight into the ways in which HCPs interpreted events and experiences with Muslim women.

### Research design

This study used semi-structured qualitative interviews with twelve HCPs from a large maternity care unit located in the North-West of England. The maternity unit provides care to a diverse BAME population including Muslim women.

An interview guide was prepared by one of the authors (SH) based on a review of the initial analysis of data from Phases 1 and 2 of the study reported elsewhere [14, 16]. Topics covered participants’ awareness of other cultures and religious beliefs, specific needs of Muslim women and good practice and training regarding care for Muslim women, full interview guide available in [16]. Interview prompts encouraged participants to share their experiences and feelings about providing care for women from different ethnic groups and their knowledge about and understanding of Muslim women’s religious practices, There were also specific questions such as *‘what do you think of women fasting during their maternity journey?’* [16]. There was also an opportunity during the interview for participants to raise issues other issues that they viewed as important, not previously covered during the interview.

Interviews took place between December 2014 and June 2015 and were conducted at a place and time of participant’s choice. This was often during lunch hours, following work shifts, or scheduled meeting and within services premises. The author conducting the interviews (SH) does not have a clinical healthcare profession background which encouraged participants to provide in-depth responses and there was no taken for granted understanding. All interviews were conducted in English, audio recorded, transcribed verbatim and anonymised with pseudonyms and numbers used to protect participants’ identities. Interviews lasted approximately 60 min.

### Participants and recruitment

Prior consultation with local HCPs provided the researcher (SH) with access to potential participants [18]. Participants were recruited through a snowballing sampling approach, whereby key contacts identified and made initial contact with potential participants. Five HCPs were identified on the grounds that they had experiences of providing maternity care for Muslim women. These HCPs identified a further seven HCPs whose experiences might be valuable to the study. Other HCPs working within the designated maternity unit and with experience of providing care for Muslim women and other BAME groups were also approached. Potential participants were provided with a detailed information sheet, highlighting the aim of the study and what would be involved should they agree to take part. 12 participants agreed to take part and signed a consent form prior to interview (Table 1).

**Table 1:** Overview of participants’ characteristics

HCP- No	HCP current role	Provided care outside of the UK	Years of providing care; more (<) or less (>)than 10 years	Ethnicity
HCP-1	Community and specialist clinic* (e.g. for non-English speakers) Midwife	Yes	< 10	White British
HCP-2	Community and specialist clinic* Midwife	Yes	< 10	White British
HCP-3	Community and specialist clinic* Midwife	No	< 10	Arab
HCP-4	Specialist clinic* Midwife	No	< 10	Other White
HCP-5	Sonographer	No	< 10	White British
HCP-6	Community midwife	No	< 10	Arab
HCP-7	Gynaecology Nurse	No	< 10	Asian
HCP-8	Gynaecology Nurse	No	> 10	Black African
HCP-9	Midwife - Labour unit	No	< 10	Asian
HCP-10	Midwife and Breast Feeding Infant Support Worker	No	< 10	White British
HCP-11	Breast Feeding Support Worker	No	> 10	Mixed (White)
HCP-12	Breast Feeding Support Worker	No	> 10	White British

### Data analysis

Data was analysed using a structured thematic analysis approach [19] to identify, analyse and report key themes within the data. Audio-recordings were transcribed verbatim by (SH) and reviewed by (CL) and (JR) for consistency and credibility.

Excel and Word software were used to manage and analyse the data. Following each interview, (SH) made notes of potential codes from each transcript, the codes were then combined and arranged into initial themes by grouping similar sets of codes together [19]. Initial themes went through various iterations, with some emerging as main themes and others sub-themes. Through a process of constant comparison [17] cycling between developing themes and transcript data, (SH) ensured that main themes reflected views expressed across several transcripts. Main themes were further confirmed through group discussion between (SH), (CL) and (JR).

The process of early data analysis and constant comparison helped to confirm that a point of data saturation had been reach, more recently described as information power [20]. Confirming that sufficient numbers of HCPs had been sampled to ensure depth and breadth of information relevant to the study’s question [20]. This processes supported the credibility of the study’s main findings.

### Ethical consideration

Ethics approval for this study was obtained from the NHS Research Ethics Committee (through the Integrated Research Application System (IRAS) [REC reference: 13/WS/0087; IRAS project ID: 117529].

Each participant was given the opportunity to read the information sheet and discuss further with (SH). Written informed consent was obtained from each participant prior to interviewing, anonymity of data and participant’s right to withdraw from the study at any point was emphasised.

## Results

Four themes were identified: 1) HCPs perceptions about Muslim women; 2) HCPs understanding and awareness of religious practices; 3) HCPs approaches in addressing and supporting Muslim women’s religious needs; 4) Importance of training in providing culturally and religiously appropriate woman-centred care.

### HCPs’ perceptions about Muslim women

When asked about Muslim women in general, participants frequently referred to there being a language barrier. In talking about their interactions, participants had a tendency to focus on their interactions with non-English speaking Muslim women and not those with English speaking Muslim women. For example, they spoke about not being able to communicate directly with the women, having instead to communicate through a mediator (interpreter or English speaking family member) – which was a great concern to all HCPs in the study. Two participants suggested that ‘*Muslim women need to learn English*’ to improve their experience of maternity care.

This was qualified by several participants who reported encounters where they had been concerned about the extent to which, Muslim women were making their own clinical decisions when they attended with their husbands. These participants noted that at times, they found Muslim women to appear to be shy, remarking how some husbands’ still communicated on behalf of their wives even when an interpreter was present. For some participants, this made them feel that the husband may be the decision maker, which they found challenging, especially in emergency situations requiring quick decisions or informed decisions on the part of both HCPs and women:

“*It does annoy me when the men interfere because then I think it is very difficult to find out [what] women actually feel like … when you have the man saying ‘no, you are not doing this to my wife” (***HCP-4 – Other White***)*

*“I have said to the interpreter, you are not speaking to the lady, you must speak my words to her. It made no difference because she (Muslim lady) looked at him (husband) for advice”* (**HCP-5- White British**)

Some participants noted that the language barrier meant that non-English speaking Muslim women had little option but to depend on English-speaking family members to communicate on their behalf and this was often the husband. Bilingual HCPs reported that they acknowledged that women with a language barrier would appreciate the opportunity to be communicated to directly and consequently often tried to communicate with women directly:

*“I speak to them directly in a language they can speak and understand, I tend to have my conversation with the woman, and the man would be asking his thing and they kind of leave me and the woman to ourselves” (****HCP-3 – Arab- bilingual****)*

Participants noted that often Muslim women depended on their family members, such as mother, grandmother, mother-in-law, aunties, sisters and cousins, for support. This included physical support during pregnancy, labour, post-labour (for up to 40 days) and in initiating and maintaining breastfeeding. More than half of participants believed that Muslim women were in an environment where they have engaged in or witnessed motherhood through the experience of other family members.

*“They [Muslim women] learn from their mothers and aunties and sisters, things like breastfeeding, they [Muslim women] just automatically will breastfeed their babies and do it well” (***HCP-1- White British***)*

However, a number of participants also mentioned how important it was for them as professionals not to make assumptions that ‘*all Muslim women are supported’* by their family, as having a big family does not necessarily mean that a woman is supported or will require less support from HCPs.

*“You should never make that assumption that all Muslim women are supported. Because even someone who has got a large family can be really isolated in that family” (***HCP-6 – Arab- Bilingual**)

### HCPs’ understanding and awareness of religious practices

Some participants showed a vague understanding of Muslim women’s religious practices during pregnancy and childbirth. A few reported on encounters where they have witnessed Muslim women observing religious practices during their care. For example, reciting the Quran (central Islamic sacred book) during their stay in hospital or whispering of the *Adhan* (Islamic call to prayer) to the new born child, which they considered to be a form of chanting without knowing specifically what this meant. However, one practice that participants were more aware of was Islamic practice of covering or modesty, which they believed often influenced Muslim women’s preference for not exposing certain parts of their body to a male professional or at times to female professionals or other women:

*“I am aware Muslim ladies are supposed to cover up and they are not meant to expose certain bits. I can tell that some Muslim women do feel uncomfortable in the hospital environment. There is only a curtain, and people do just pop in”* (**HCP-11- Mixed White**)

More than half of participants reported that they were aware of the Islamic teaching that gives Muslim women the option to be attended to by a male HCP where a female HCP is unavailable. However, participants found that not all Muslim women would consider this option. A number of participants reported encounters where Muslim women had declined a male HCP even in critical or emergency situations. Some participants reported how they found it difficult to understand why some Muslim women would not take the religious flexibility permitted to be attended to by a male HPC especially when faced with an emergency:

*“[Previously] I was certain that Muslim women would judge the situation [a critical or not critical] appropriately [when making the decision about their gender preference of the HCP]. [However] one scenario where a Muslim woman did not want a male doctor. She was bleeding internally, she had an accident but would not let a male doctor touch her. I think perhaps most people would think that would be horrendously stupid”* (**HCP-5 – White British**)

Participants also discussed Muslim women’s practice of fasting during the month of Ramadhan. While most participants were aware that pregnant and breastfeeding women are exempt from fasting, they found that most Muslim women either attempted to fast the entire month or at least a few days. Participants reported that it was difficult to provide advice, especially when women were medically unfit to undertake fasting. Participants described occasions when some Muslim women had not informed a HCPs of their fasting practice, which made it even more challenging to advise or discuss the concept of fasting during pregnancy and breastfeeding.

*“I cannot ever recall [fasting] being a key topic … They might say, I feel a bit faint or I feel very tired, but they would never link it with fasting [laugh] until you delve a bit deeper or say, are you fasting? Then that information comes out” (***HCP-4 – Other White***)*

*“‘I do not think you can ever be so direct because then you are hitting a line, where she [Muslim woman] thinks you are judging her and you are being rude. I think it is always about asking, have you been fasting?” (***HCP-7 - Asian-bilingual***)*

Participants described other religious practice that they found to influence Muslim women opinions and decisions on certain medical interventions, such as screening for Down Syndrome (DS). Participants reported that often Muslim women would refuse or only complete the initial stage (ultrasound scan and blood test for DNA markers) of DS screening due to their religious injunctions on terminating a pregnancy except in situations where the mother’s life is at risk. The DS screening gives women a diagnosis if their unborn baby has DS, the options to following this includes continue with the pregnancy and prepare for their child with the condition, or to terminate the pregnancy. Participants found that due to the option to terminate the pregnancy, most Muslim women would not give much thought about having screening that could help them prepare for if they did receive a diagnosed of DS, instead, quickly choosing to decline DS screening.

Participants also explained that following the initial stage of the DS screening women at high risk are offered further diagnostic tests involving amniocentesis or Chorionic villus sampling where a fine needle (usually inserted through the mother’s abdomen, is used to take a sample of tissue from the placenta or fluid surrounding the baby). Participants reported that when explaining these procedures to Muslim women and the risk of about 0.5 to 1 in 100 chance that these diagnostic tests result in a miscarriage, most Muslim women would refuse the test out of fear of the risk of miscarriage. Although HCPs reported that the majority of Muslim women tended to decline the DS screening, this did not prevent them from offering the screening to all Muslim women.

HCPs in this study were familiar with the Arabic term ‘*Inshallah*’ meaning ‘in God’s will’ and they reported that they had encountered Muslim women using the term when refusing DS screening or refusing to go through with a planned or emergency caesarean section. Some HCPs found this Islamic concept of fate challenging at times for example, when an emergency caesarean section is required or when it is the only safe option for the women:

*“She [Muslim woman] - not her husband - she would not consent to a [emergency] caesarean section because, it was inshallah (God's will). I saw that also in Saudi that sometimes the woman would refuse a caesarean section and it was ‘inshallah’ … Even when we explained everything, how the babies might die, she [Muslim woman] was still it was God's will … husband actually persuaded her to have the caesarean section. Inshallah is said a lot in Saudi Arabia and it is said a lot here too”* (**HCP-1- White British**)

Participants acknowledged the importance of giving women choice, however, they felt that when a woman’s health is at risk, it was often difficult to appropriately honour decisions for example, when a Muslim women refused an emergency caesarean due to the gender (male) of the HCP that would carry out the caesarean section. Some participants reported that once Muslim women understood the benefits and the risks of a caesarean section, they often gave their consent. However, HCPs in this study mentioned that they found it very challenging where women remain resistant in such situations:

*“It is very difficult, because you are trying to give women choice but you know that they would not be able to deliver a baby naturally. I think you just have to keep plodding away and explaining why, and give the good reasons why women need that procedure. It is been very difficult really”* (**HCP-4 –Other White**)

### HCPs approaches in addressing and supporting Muslim women’s religious needs

Overall, participants reported that they would not treat one woman any differently to any other woman, but would treat all women as individuals with unique needs. However, participants felt that ensuring women understand reasons for care was fundamental for high quality care, especially for women where there was a language barrier. Participants used available services such as face-to-face interpreters, language line (telephone interpretation services), and Health Link workers (includes bilingual workers that support face-to-face interpretation) to help women understand their care and the implications of clinical decisions. Participants also sought the support of bilingual colleagues to communicate reliable information. Four participants found that having access to midwives who were bilingual enabling them to communicate with the women without having to use interpreters, had been of great benefit to delivering high quality care:

*“I think it is great having an Arabic speaking midwife in the clinic, because women do not need that third person. I think it helps with women's perception about what we are trying to offer them in terms of healthcare in the community. I think we should be embrace the use of bilingual staff and try to encourage it more”* (***HCP-4- Other White***)

A few participants were keen on learning universal Arabic terms such as ‘*Inshallah*’ (In Gods will) or the greeting ‘*As-salamu alaykum’* (peace be upon you) that are used by Muslims in general, to enhance communication and build mutual relationships with Muslim women. Participants found that using such words acted as an icebreaker that helped Muslim women feel comfortable and relaxed during their care.

The majority of participants mentioned that they would provide a private room or have the curtains drawn if preferred by the Muslim women, for example while breastfeeding. Participants also reported that they were mindful of Muslim women who wear the Hijab (headscarf) or who do not expose certain parts of their body and they would for example, provide extra hospital gowns or sheets to help these Muslim woman to be covered appropriately while in a theatre gown prior to being taken to theatre:

*“When Muslim women are in the little hospital gowns, they stay in bed all day, and people [HCP] do not understand why they are in bed when they should be walking around. It is probably because they [Muslim women] have not got anything to cover their legs. So I get them [Muslim women] stockings or something just to put on their legs, or just look for something to cover their legs”* (**HCP-7 - Asian)**

Finally, overall participants in this study found that the deeper understanding they had of Islam and other faiths the more sensitive they were able to be to the needs of women of all faiths. For example, half of the participants provided Muslim women with a CD player to play the recitation of the Quran during their stay in hospital and one Muslim HCP reported printing Islamic supplications that are recommended for Muslim women to read while in labour.

### Importance of training in providing culturally and religiously appropriate woman-centred care

Participants discussed training methods that might bring about awareness of, and promote competency in addressing the needs of diverse populations. Participants felt that the current training offered did not equip them with the knowledge and skills to address the needs of diverse populations with specific cultural or religious aspects linked to maternity care:

*“Culture and religion is a big thing, and people do not really seem to understand that there is a culture and there is a religion, and they are not the same. I think that is something that needs to be identified; training should help highlight the difference between culture and religion*” (**HCP-7 – Asian)**

Participants discussed methods that they believe might be effective in promoting wider cultural and religious awareness amongst HCPs that reflected the needs of women from different diverse backgrounds. Participants in particular, were not in favour of individual online training that consists of multiple-choice questions which they found to be ineffective in enabling understanding and provoking discussion. Instead participants preferred training methods that promoted group discussion and sharing of experiences - giving HCPs the chance to question and learn from their daily experiences. Participants were keen to attend such training if this was provided within their Trust:

*“I think they need to look at training and maybe revamp it and make it more in-depth and address it to what we deal with on a daily basis, rather than a Human Rights Act and ten questions online about general equality and diversity. Because if somebody who was Jewish came, I would not have a clue what her beliefs are, what would help her or how to deal with anything special”* (**HCP-8- Black African - bilingual**)

HCPs in this study in this study suggested that building cultural knowledge through experiences and encounter with women from different backgrounds is important, consequently opportunities to work or train within a clinic that specifically focus on providing care for BAME groups (such as clinics for non-English speakers) was welcomed by HCPs:

*“In the [name] clinic you could see ten women with ten different languages, from ten different cultural backgrounds, in one morning. Therefore, to me, much more powerful teaching tool, than an online book or even me sitting talking about it in a classroom”* (**HCP-4 – Other White**)

HCPs in this study highlighted the nature of their demanding work environment and how having to attend additional training could potentially increase their workload without really giving them much benefit. However, participants indicated that if training was available that involves real life-birth experiences being told by the women themselves they would be more enthusiastic about attending and would benefit greatly.

Finally, Participants noted that it was essential to give more focus to cultural and religious training as part of university courses for various HCPs. They suggested that educational institutions should further equip students with knowledge, skills, and openness to continuous self-development. They suggested that training should include different aspects of cultural and religious values, recognising diversity in care provision for diverse populations to promote and ensure inclusivity in service provision.

## Discussion

This paper presents the experiences of 12 HCPs providing maternity services for Muslim women in the UK. In this study, HCPs reported the different challenges they faced whilst dealing with Muslim women’s needs and religious practices. Participants highlighted the need for training that specifically focuses on enhancing knowledge and understanding of the different cultural and religious values and practices that influence healthcare choices. Our findings suggest that training for HCPs should cover aspects such as; preconception about Muslim women that impact of provision of health care; religious practices that may impact on the effectiveness of clinical care (such as fasting during pregnancy); examples from those with lived experience of delivering appropriate high quality care; and other practices that may enhance the maternity experience such as attending to Islamic modesty and covering/dress code.

In the current study, many HCPs reported that they had developed some awareness of Muslim women’s needs and religious practices through their face-to-face interactions with Muslim women. Although HCPs appeared to be aware of certain religious needs, lack of clear definitions and practical guidance often left HCPs uncertain regarding how they might address certain religious needs alongside delivering effective clinical care [21]. It was apparent that awareness and understanding of the needs of different groups of women from diverse BAME backgrounds extends far beyond a basic understanding of language needs [22]. Optimal care also required an understanding and recognition of individual cultural differences and the barriers or biases that are unconsciously created by HCPs and the importance of cultural for childbearing women [22]. Such cultural and religious awareness and understanding is vital in healthcare services - a better understanding of Muslim women’s religious needs will not only equip HCPs with important skills so they can provide appropriate care for women that is not only women-centred but also sensitive to cultural and religious difference creating a mechanism to bridge the current gap in communication between HCPs and women [14, 22, 23].

The Code for Nurses and Midwives (2015) advises that nurses and midwives keep their knowledge and skills up to date, by taking part in appropriate and regular learning and professional development activities with that aim of maintaining and developing one’s professional competence and improve one’s performance [24]. Woman-centred care requires a flexible and collaborative care model that both respects and accommodates the needs of all women [25].

While HCPs may be aware of the importance of cultural or religious awareness and competency, they may not necessarily have access to cultural or religious knowledge, to inform their practice. One study reported that HCPs who received cultural competence training, demonstrated greater understanding of the central role of culture in healthcare and recognised common barriers to cultural understanding among providers, staff and service users [26]. Cultural or religious awareness also enhances HCPs understanding and recognition of individual differences, and removes barriers that are unconsciously created due to lack of awareness of the importance of women’s value systems [22].

Consistent with findings from other studies, HCPs in our study suggested that they would benefit from training that incorporated women’s lived experiences and helped to provoke discussion [14, 27]. They recommended that training and resources should be facilitated in a way that enhanced their knowledge and skills in providing culturally and religiously appropriate woman-centred care. Strategies such as e-learning were not perceived as necessarily the best way to connect professionals with the voices and feelings of women [28]. Birth narratives can provide insights about the connection between childbearing and different value systems, and can be utilised as an effective intervention for childbirth educators [27].

### Strengths and limitations

This is the first qualitative study that explored the experiences of HCPs providing care for Muslim women in the North West of the UK. The findings provide valuable insights into the experiences and challenges of HCPs whilst addressing the maternity care needs of Muslim women. Participants’ narratives provided a rich and contextualised account of their experiences, providing insight that that enhances our understanding of Muslim women needs in relation to maternity care. Our findings will enable the further development of training for HCPs to support the development of literacy and competency skills necessary in addressing the needs of Muslim women.

The main limitation of this study is the recruitment approach. Participants were recruited from one maternity care unit operating within an ethnically diverse area in the North West of England, which caters for the needs of a significant proportion of Muslim women. Therefore, HCPs in this study may have a higher level of cultural competency than HCPs working in less ethnically diverse areas. In addition, just under half of the sample were Muslim HCPs who acted as cultural brokers for Muslim women accessing maternity services. Having Muslim participants with an in-depth understanding of religious practices, may have limited the ability of the study to reflect on the challenges faced by HCPs while providing care for Muslim women.

Finally, the snowball sampling approach may have further limited outreach to other potential participants. Other possible approaches to recruitment in future studies might include recruitment of HCPs through General Practitioner surgeries, children centres, family planning clinics, or advertisements through the Trust’s main website.

## Conclusion

This study makes an important contribution to the wider understanding of HCPs’ experiences whilst providing maternity care for Muslim women in the UK. All HCPs in this study were keen to provide care that best meets the care needs of Muslim women, and highlighted the importance of effective communication in doing so. However, a lack of appropriate training to support HCPs when providing culturally and religiously appropriate woman-centred care for Muslim women was highlighted.

This study highlights the importance of specific cultural and religious knowledge including Muslim women’s practices and the urgent need for training to support the development of such knowledge amongst HCPs. Such training extends far beyond understanding of language needs and lists of ‘dos’ and ‘do nots’ or facts about other value systems, requiring in addition, an emphasis on care that acknowledges, accepts and responds to individual differences, helping to create an atmosphere where all women feel able to express and discuss their specific healthcare needs.

## Data Availability

Qualitative data extracts are presented in the article to support the findings. The original transcripts are not available to the public as they may contain information that could compromise the confidentiality and anonymity of the participants.

## Abbreviations

HCPs: Healthcare Professionals
BAME: Black, Asain and Minority Ethnicity
DS: Down Syndrome screening
IRAS: Integrated Research Application System
